# Dysregulation of miR-15a and miR-214 in human pancreatic cancer

**DOI:** 10.1186/1756-8722-3-46

**Published:** 2010-11-24

**Authors:** Xing J Zhang, Hua Ye, Cheng W Zeng, Bo He, Hua Zhang, Yue Q Chen

**Affiliations:** 1 Key Laboratory of Gene Engineering of the Ministry of Education, State Key Laboratory for Biocontrol, Sun Yat-sen University, Guangzhou 510275, PR China; 2 The Second Affiliated Hospital of Sun Yat-sen University, Guangzhou, 510120, PR China

## Abstract

**Background:**

Recent reports indicate that microRNAs (miRNAs) play a critical role in malignancies. However, the role that miRNAs play in pancreatic cancer remains to be determined. The purpose of this study was to investigate aberrantly expressed miRNAs in pancreatic cancer tissues and demonstrate their roles in disease progression.

**Results:**

We detected the expression patterns of miRNAs in 10 pancreatic cancer tissues and their adjacent benign tissues by quantitative real time-PCR (qRT-PCR) and found that miR-15a and miR-214 were dysregulated in the tumor samples. This is the first time that miR-214 has been identified as aberrantly expressed in pancreatic cancer. In vitro experiments showed that overexpression of miR-15a inhibited the viability of pancreatic cancer cells, whereas overexpression of miR-214 decreased the sensitivity of the cells to gemcitabine (GEM). Furthermore, we identified WNT3A and FGF7 as potential targets of miR-15a and ING4 as a target of miR-214.

**Conclusions:**

Aberrant expression of miRNAs such as miR-15a and miR-214 results in different cellular effects in pancreatic cancer. Downregulation of miR-15a might contribute to proliferation of pancreatic cancer cells, whereas upregulation of miR-214 in pancreatic cancer specimens might be related to the poor response of pancreatic cancer cells to chemotherapy. MiR-15a directly targets multiple genes relevant in pancreatic cancer, suggesting that it may serve as a novel therapeutic target for treatment of the disease.

## Background

Pancreatic cancer is a disease with a high rate of mortality. It is generally diagnosed at an advanced stage, at which point no successful therapies are available. Pancreatic cancer is characterized by the potential for local invasion, enabling it to spread during early developmental stages of the disease. Even when diagnosed early, the limited response of pancreatic cancer to available treatments, including surgical resection and chemotherapeutics, contributes to its high mortality rate [1,2]. Therefore, there is an urgent need to discover novel early diagnostic biomarkers and to identify new therapeutic strategies. However, the molecular mechanisms underlying the high tumorigenicity of pancreatic cancer are not well known.

Recently, a new family of small regulatory RNAs called microRNAs (miRNAs) was discovered, and their roles in many biological processes are under investigation. MiRNAs are short (approximately 22 nt in length) noncoding RNAs that regulate gene expression [3] and have been implicated in the regulation of cancer progression [4-6]. By negatively regulating tumor suppressor genes or oncogenes, miRNAs can play a role in promoting cancer [5].

Unlike most currently available biomarkers, miRNA expression appears to be cell type- and disease-specific and can be used for the classification of certain cancer histotypes [7,8]. Various miRNAs are aberrantly expressed in pancreatic cancer, and these aberrant expression patterns can accurately differentiate pancreatic cancer from benign pancreatic tissues [9-12]. Lee et al. also identified several miRNAs aberrantly expressed in pancreatic ductal adenocarcinoma (PDAC), which suggests that these novel molecules could serve as diagnostic biomarkers for the disease [13]. However, the association between miRNAs and their roles in pancreatic cancer progression remains to be elucidated.

In this study, we demonstrated that miR-15a and miR-214 were significantly dysregulated in pancreatic cancer specimens. MiR-15a was frequently downregulated in the cancer samples relative to the benign tissues samples, whereas miR-214 was upregulated. In pancreatic cancer, miR-15a directly regulates WNT3A and FGF7, and miR-214 might regulate ING4. In addition, we found that overexpression of miR-15a could reduce the viability of pancreatic cancer cells, whereas miR-214 counteracted the pro-apoptotic effect of gemcitabine (GEM) in BxCP-3 cells.

## Results and discussion

### MiR-15a downregulation and miR-214 upregulation in human pancreatic cancer

To identify dysregulated miRNAs, we used qRT-PCR to measure the expression of seven mature miRNAs (miR-15a, miR-27a, miR-100, miR-125b, miR-181a, miR-200a and miR-214) in 10 pancreatic cancer tissues and their adjacent benign tissues. These seven mature miRNAs were chosen based on recent reports that identified them as having important functions in cancers. After normalization to U6 RNA expression as a control, the differential expression patterns of the miRNAs in cancer and benign pancreatic tissues were determined.

**Figure 1:**
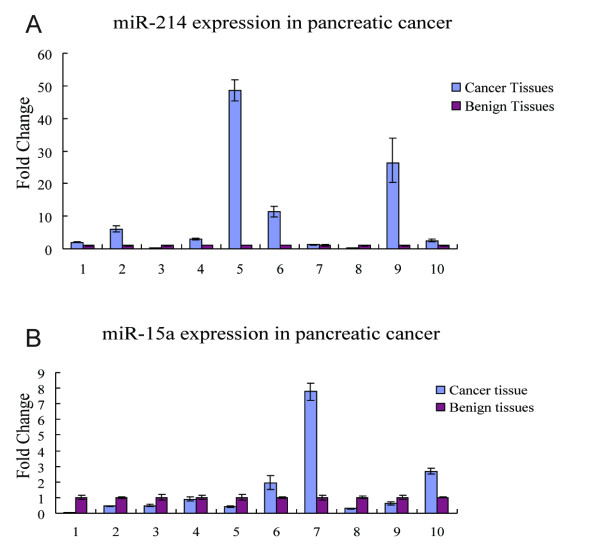
**Expression patterns of miR-15a and miR-214**. qRT-PCR was performed to detect (A) miR-214 and (B) miR-15a expression in 10 pancreatic cancer tissues and their adjacent benign pancreatic tissues. Expression levels of miRNAs in adjacent benign pancreatic tissues were set as 1. Relative values were calculated to indicate the frequency of miRNA expression downregulated or upregulated in pancreatic cancer.

Among the miRNAs studied, we found that four miRNAs were frequently overexpressed in the cancer tissues studied: miR-100, miR-125b, miR-200a and miR-214 (Table 1 and Figure 1A). In particular, miR-214 expression was elevated in 8 of 10 (80%) cancer specimens. Only one miRNA, miR-15a, showed decreased expression in cancer tissues compared with matched benign pancreatic tissues; this effect was evident in 7 of 10 (70%) samples (Figure 1B).

**Table 1:** Expression of miRNAs in pancreatic cancer specimens compared with adjacent benign pancreatic tissues

miRNA	Median valve	Upregulated in pancreatic cancer reference (%)
miR-15a	0.56	(30%)
miR-27a	1.27	(50%)
miR-100	3.29	(70%)
miR-125b	3.16	(70%)
miR-181a	0.96	(50%)
miR-200a	2.78	(70%)
miR-214	2.78	(80%)

qRT-PCR was used to measure expression of seven miRNAs in 10 pancreatic cancer tissues and their adjacent benign pancreatic tissues. MiRNA expression levels are represented as relative values, compared to those of adjacent benign pancreatic tissues, which were taken as 1. Median value was calculated to indicate the frequency of a miRNA expression downregulated or upregulated in pancreatic cancer.

Among the four upregulated miRNAs, miR-214 was previously reported to be associated with mouse pancreas development [14]. However, there are no reports on the function of miR-214 in human pancreas development or in the chemoresistance of pancreatic cancer. This is the first report implicating the dysregulation of miR-214 in pancreatic cancer. As for miR-15a, a tumor suppressor that has been reported in various cancers, its functions in pancreatic cancer are unknown; however, it was the only one downregulated in our examination. Therefore, miR-214 and miR-15a were chosen for further study.

### MiR-15a overexpression reduces cell viability, whereas miR-214 decreases sensitivity to GEM in pancreatic cancer cells

**Figure 2:**
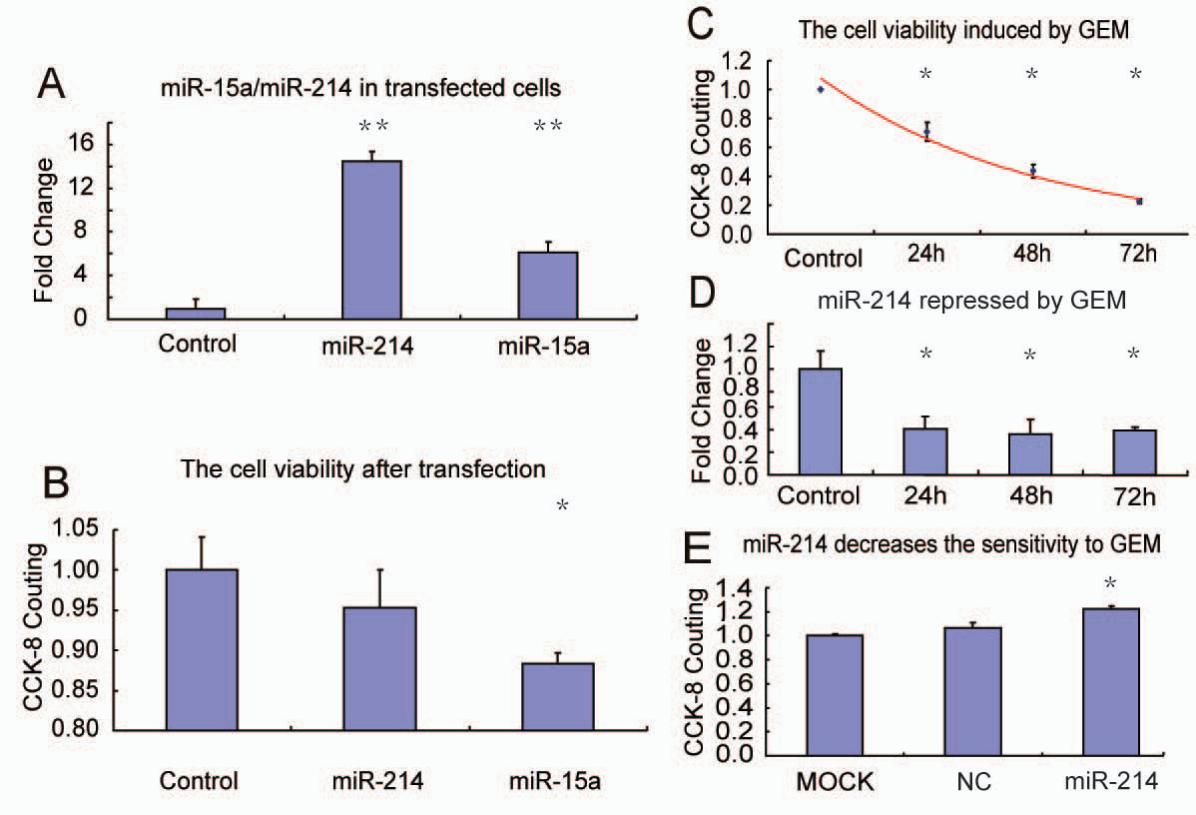
**MiR-15a and miR-214 have different roles in pancreatic cancer cells**. (A) qRT-PCR was used to investigate miRNA transfection efficiency. Both miR-15a and miR-214 were significantly increased compared to their mimics-NC (control) in BxCP-3 cells. (B) The viability of BxCP-3 cells after transfection was measured by CCK-8 assay. (C) Cell viability was measured using the CCK-8 assay in BxCP-3 cells treated with 10 μM GEM at 24, 48 and 72 hrs. (D) The expression pattern of miR-214 was detected by qRT-PCR in BxCP-3 cells treated with GEM. (E) The CCK-8 assay was used to measure the inhibition effect of miR-214 on apoptosis of BxCP-3 cells induced by GEM. BxCP-3 cells were transfected with H_2_O (MOCK), mimics-NC (NC), and miR-214 mimics (miR-214). Significant differences (* p < 0.05; ** p < 0.01) compared with the control were calculated using Dunnett's test or the paired Student's *t*-test.

To investigate the potential functions of miR-15a and miR-214 in pancreatic cancer, we first measured the viability of cells transfected with miR-15a/miR-214 mimics or their controls (mimics-NC) using the CCK-8 assay. BxCP-3 pancreatic cells were used in our examination. The transfection efficiency of both miR-15a and miR-214 and their corresponding controls in BxCP-3 cells was measured by qRT-PCR assay. The results were analyzed using the paired Student's *t*-test. MiR-214 was upregulated more than 14-fold in BxCP-3 cells after transfection, whereas miR-15a was upregulated about 6-fold (Figure 2A); this result indicated better transfection efficiency of miR-214. We then assessed cell viability. The CCK-8 assay showed that overexpression of miR-15a significantly decreased the viability of BxCP-3 cells compared with the control (p < 0.05) (Figure 2B). These results indicate that the expression level of miR-15a is important for pancreatic cancer cell growth. Because miR-15a was downregulated in pancreatic cancer, we hypothesized that miR-15a might function as a tumor suppressor in the disease, a role it has been shown to play in other cancers [15-18].

Documented evidence indicates that miR-214 functions as either an oncogene or a tumor suppressor in different cancers. It was also reported that miR-214 negatively regulates HeLa cell proliferation and increases the ability of T cells' viability [19,20]. However, we observed no obvious effect of miR-214 overexpression on cell viability (p > 0.05) (Figure 2B), which implies that miR-214 might have other roles in pancreatic cancer. A previous study showed that miR-214 can promote cell survival and cisplatin resistance in human ovarian cancer [21]. Because overexpression of miR-214 was observed in pancreatic cancer tissues, we questioned whether this phenomenon might be related to tumor cell survival and drug resistance in pancreatic cancer.

To address this issue, we investigated the expression patterns of miR-214 in BxCP-3 cells treated with GEM. GEM is currently the first-line treatment for advanced pancreatic cancer, and it acts by inhibiting tumor cell proliferation and inducing apoptosis [22-25]. Prior to determining the effects of GEM on miR-214, we examined the effect of GEM on cell viability at 24, 48 and 72 hrs using the CCK-8 assay. Cell viability decreased in a time-dependent manner in response to GEM treatment (Figure 2C). After 72 hrs of 10 μM GEM treatment, cell viability decreased to approximately 20% compared with untreated cells. Next, we detected the expression pattern of miR-214 in cells treated with GEM. We found that miR-214 was dramatically downregulated after treatment with GEM. MiR-214 levels decreased by 60% at 24 hrs and remained low for 72 hrs (Figure 2D), indicating that miR-214 was responding to the drug treatment. We then investigated whether overexpression of miR-214 could modulate the sensitivity of BxCP-3 cells to GEM-induced apoptosis. After 72 hrs of GEM treatment, we found that the viability of BxCP-3 cells transfected with miR-214 mimics was significantly higher (about 22%) than that of the NC and MOCK negative control groups (Figure 2E). These results suggest that miR-214 might be involved in the chemoresistance of pancreatic cancer cells.

### MiR-15a suppresses cell viability by regulating WNT3A and FGF7, and miR-214 potentially downregulates ING4 to inhibit apoptosis induced by GEM

**Figure 3:**
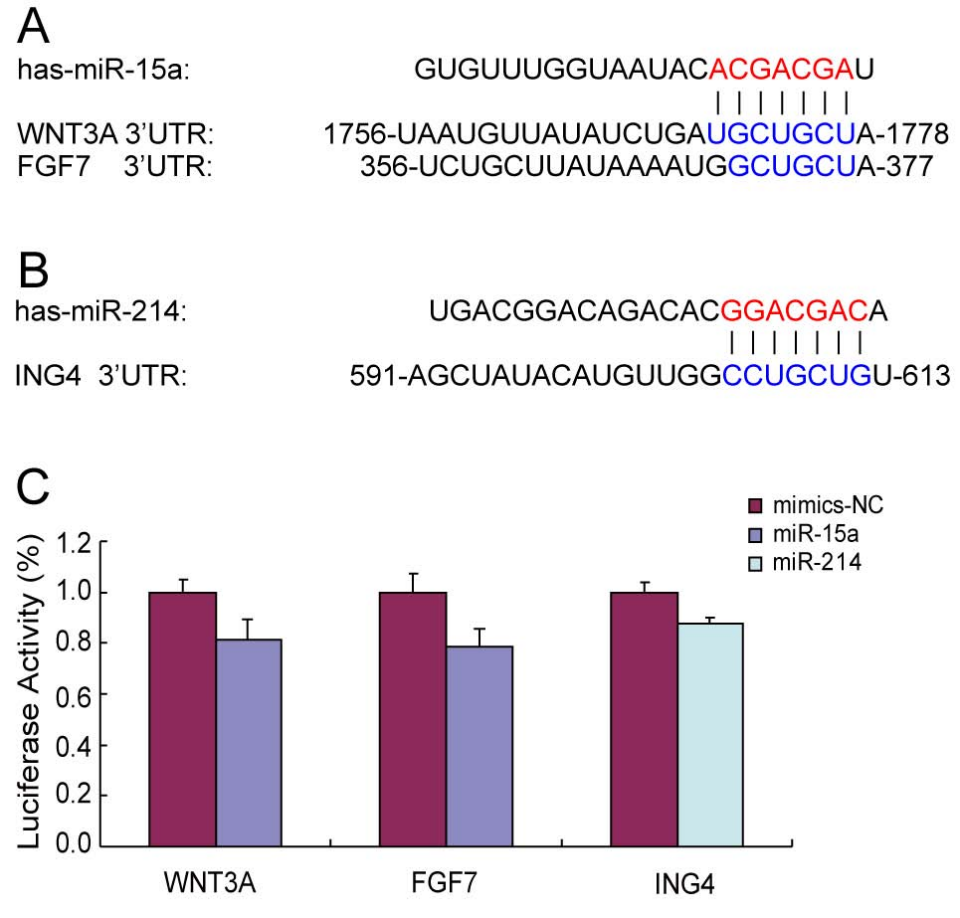
**Target validation of miR-15a and miR-214**. (A) The 3'-UTR of WNT3A and FGF7 contain predicted MREs for miR-15a. (B) The 3'-UTR of ING4 contains the predicted MRE for miR-214. (C) A luciferase assay was used to measure the activity of the 3'-UTR reporter in 293T cells. MiR-15a inhibited the activity of WNT3A and FGF7 3'-UTR reporters, whereas miR-214 inhibited the activity of the ING4 3'-UTR reporter.

To further study the mechanisms of both miR-15a and miR-214 in pancreatic cancer cells, we predicted and validated potential targets for both miRNAs. Putative target genes that were identified by one or more of five different target prediction algorithms (PicTar, TargetBoost, TargetScanS, MiRanda and miRbase) were screened for the location and number of putative binding sites as well as their biologic relevance. Among the candidate targets of miR-15a chosen for experimental validation were PIM1, CDC25A, BCL2L2, WNT3A, SMAD7, LRP6 and FGF7, each of which has been reported to play a role in cell proliferation (Table 2). Using the same methods, seven candidates: RASSF5, PIM1, BAX, BIK, NEO1, ACVR1B and ING4, were predicted as the putative targets of miR-214 and chosen for experimental validation (Table 3). The wild-type 3'-UTR of each gene was cloned into the 3'-UTR of a Renilla luciferase reporter gene of a modified psiCHECK2 expression vector, and the resultant constructs were transfected into 293T cells using Lipofectamine 2000. Luciferase expression in cells expressing the WNT3A and FGF7 reporters was significantly suppressed (18% and 20%, respectively) when co-transfected with miR-15a mimics (Figure 3A and 3C). These data indicate that WNT3A and FGF7 might be targets of miR-15a. In addition, miR-214 repressed the luciferase activity of the ING4 reporter construct by 13% (Figure 3B and 3C). Expression levels of the remaining reporter constructs were unaffected by miRNA co-transfection.

**Table 2:** Target validation for miR-15a

miR-15a target		Synthesized 3'-UTR containing the predicted MRE	MRE validated by luciferase activity	Specifically suppressed by miR-15a mimics
PIM1	F	TCGAGTACTTGAACTTGCCTCTTTTACCTGCTGCTTCTCCAAAAATCTGCCTGGGTTGC	YES	NT
			
	R	GGCCGCAACCCAGGCAGATTTTTGGAGAAGCAGCAGGTAAAAGAGGCAAGTTCAAGTAC		

CDC25A	F	TCGAGGAGTAGAGAAGTTACACAGAAATGCTGCTGGCCAAATAGCAAAGACAACCTGGC	YES	NT
			
	R	GGCCGCCAGGTTGTCTTTGCTATTTGGCCAGCAGCATTTCTGTGTAACTTCTCTACTCC		

BCL2L2	F	TCGAGGATTTTATTTGCATTAAGGGGTTTGCTGCTGAAAAAAAGTTGGAAAACCACTGC	YES	NT
			
	R	GGCCGCAGTGGTTTTCCAACTTTTTTTCAGCAGCAAACCCCTTAATGCAAATAAAATCC		

WNT3A	F	TCGAGCGTTTTTGGTTTTAATGTTATATCTGATGCTGCTATATCCACTGTCCAACGGGC	YES	YES
			
	R	GGCCGCCCGTTGGACAGTGGATATAGCAGCATCAGATATAACATTAAAACCAAAAACGC		

SMAD7	F	TCGAGCAGGCCACACTTCAAACTACTTTGCTGCTAATATTTTCCTCCTGAGTGCTTGGC	YES	NT
			
	R	GGCCGCCAAGCACTCAGGAGGAAAATATTAGCAGCAAAGTAGTTTGAAGTGTGGCCTGC		

LRP6	F	TCGAGTATATATTTTCTTAAAACAGCAGATTTGCTGCTTGTGCCATAAAAGTTTGTAGC	YES	NT
			
	R	GGCCGCTACAAACTTTTATGGCACAAGCAGCAAATCTGCTGTTTTAAGAAAATATATAC		

FGF7	F	TCGAGTATTCCTATCTGCTTATAAAATGGCTGCTATAATAATAATAATACAGATGTTGC	YES	YES
			
	R	GGCCGCAACATCTGTATTATTATTATTATAGCAGCCATTTTATAAGCAGATAGGAATAC		

The 59-bp segments of the 3'-UTR of each target gene are listed in this table. F (forward sequence) and R (reverse sequence) were annealed together and inserted into the psi-CHECK-control vector. NT, negative.

**Table 3:** Target validation for miR-214

miR-214 target		Synthesized 3'-UTR containing the predicted MRE	MRE validated by luciferase activity	Specially suppressed by miR-214 mimics
PIM1	F	TCGAGTACTTGAACTTGCCTCTTTTACCTGCTGCTTCTCCAAAAATCTGCCTGGGTTGC	YES	NT
			
	R	GGCCGCAACCCAGGCAGATTTTTGGAGAAGCAGCAGGTAAAAGAGGCAAGTTCAAGTAC		

RASSF5	F	TCGAGCTCCCTTTAGAAACTCTCTCCCTGCTGTATATTAAAGGGAGCAGGTGGAGAGC	YES	NT
			
	R	GGCCGCTCTCCACCTGCTCCCTTTAATATACAGCAGGGAGAGAGTTTCTAAAGGGAGC		

BAX	F	TCGAGTGATCAATCCCCGATTCATCTACCCTGCTGACCTCCCAGTGACCCCTGACCTGC	YES	NT
			
	R	GGCCGCAGGTCAGGGGTCACTGGGAGGTCAGCAGGGTAGATGAATCGGGGATTGATCAC		

BIK	F	TCGAGACCACTGCCCTGGAGGTGGCGGCCTGCTGCTGTTATCTTTTTAACTGTTTTCGC	YES	NT
			
	R	GGCCGCGAAAACAGTTAAAAAGATAACAGCAGCAGGCCGCCACCTCCAGGGCAGTGGTC		

NEO1	F	TCGAGTGTGTCGAGGCAGCTTCCCTTTGCCTGCTGATATTCTGCAGGACTGGGCACCGC	YES	NT
			
	R	GGCCGCGGTGCCCAGTCCTGCAGAATATCAGCAGGCAAAGGGAAGCTGCCTCGACACAC		

ING4	F	TCGAGGTAAATAAAAGCTATACATGTTGGCCTGCTGTGTTTATTGTAGAGACACTGTGC	YES	YES
			
	R	GGCCGCACAGTGTCTCTACAATAAACACAGCAGGCCAACATGTATAGCTTTTATTTACC		

ACVR1B	F	TCGAGTCATTGGGGGGACCGTCTTTACCCCTGCTGACCTCCCACCTATCCGCCCTGCGC	YES	NT
			
	R	GGCCGCGCAGGGCGGATAGGTGGGAGGTCAGCAGGGGTAAAGACGGTCCCCCCAATGAC		

The 59-bp segments of the 3'-UTR of the target genes are listed in the table. F (forward sequence) and R (reverse sequence) were annealed together and inserted into the psi-CHECK-control vector. NT, negative.

WNT3A is a member of the Wnt/β-catenin signaling pathway. Dysregulated Wnt/β-catenin signaling has been linked to various human diseases, including cancer. WNT3A promotes the activation of survival and proliferation pathways through the phosphorylation of the kinases ERK and Akt. Here, we demonstrated that WNT3A may also be a direct target of miR-15a. Moreover, we identified FGF7, a fibroblast growth factor, as another potential target of miR-15a. FGF7 was reported to play an important role in pancreatic organogenesis, and FGF10/FGFR2 signaling recently emerged as a promising new molecular target for pancreatic cancer [26]. MiR-15a directly targets multiple genes relevant in pancreatic cancer and therefore may serve as a novel therapeutic target in pancreatic cancer.

The tumor suppressor ING4 belongs to the ING family of genes, which comprises type II tumor suppressor genes [27,28] involved in cell cycle arrest, transcriptional regulation, DNA repair and apoptosis. Downregulation of ING4 has been reported in various tumors, including gliomas, breast tumors and stomach adenocarcinoma. Hepatocellular carcinoma (HCC) patients with low ING4 expression had poorer overall survival and disease-free survival than those with high expression [29]. Xie et al. found that upregulation of ING4 could suppress lung carcinoma cell invasiveness and reduce tumor microvessel formation [30]. It was also reported that miR-650 targets ING4 to promote gastric cancer tumorigenicity [31]. In the present study, we found that ING4 is a potential target of miR-214, which was overexpressed in pancreatic cancer and could modulate the sensitivity to GEM-induced apoptosis in BxCP-3 cells. Expression levels of miR-214 could potentially serve as prognostic markers; however, the utility of miR-214 as a therapeutic target in human pancreatic cancer remains to be determined.

## Conclusions

MiR-15a and miR-214 were found to be aberrantly expressed in human pancreatic cancer and to play different roles in the development of the disease. Overexpression of exogenous miR-15a inhibited the viability of pancreatic cancer cells, suggesting that downregulation of miR-15a might be involved in the progression of pancreatic cancer. Moreover, we confirmed that WNT3A and FGF7 are potential targets of miR-15a. MiR-15a directly targets multiple genes relevant in pancreatic cancer, suggesting that it may serve as a novel therapeutic target in pancreatic cancer. MiR-214 is another miRNA that is dysregulated in pancreatic cancer. We found that miR-214 promoted survival of pancreatic cancer cells as well as GEM resistance, which might be related to the poor response to chemotherapy in pancreatic cancer patients. We also identified ING4 as a potential target of miR-214. The detailed mechanisms and signaling pathways regulated by miR-15a and miR-214 in pancreatic cancer deserve further study.

## Materials and methods

### Cell cultures and clinical samples

BxPC-3 human pancreatic cancer cells were maintained in RPMI 1640 medium containing 10% fetal bovine serum (FBS; Gibco BRL). 293T cells were maintained in DMEM containing 10% FBS.

Ten samples of pancreatic cancer tissues and their adjacent benign tissues were obtained from patients at the Second Affiliated Hospital of Sun Yat-sen University. All specimens were immediately snap-frozen in liquid nitrogen and stored at -80°C. Patient characteristics are available for all patients. Written informed consent for the biological studies was obtained from the patients involved in the study or from their parents/guardians. The study was approved by the Ethics Committee of the affiliated hospitals of Sun Yat-sen University.

### RNA extraction and qRT-PCR

Total RNA was isolated with Trizol (Invitrogen, Carlsbad, CA) according to the manufacturer's instructions. qRT-PCR was performed as previously described [32] using the Hairpin-it™miRNAs Real-Time PCR Quantization Kit (GenePharma, Shanghai, China) containing a stem-loop-like RT primer and PCR primers specific to the various miRNAs or the U6 RNA internal control (Table 4). The expression of miRNAs in tumor tissues relative to that in adjacent benign tissues was determined using the 2^-ΔΔCT ^method [33]. Briefly, the △C_T _of each miRNA was determined relative to that of the U6 endogenous control RNA, which was robustly and invariantly expressed across all samples. MiRNA expression levels in each of the 10 microdissected pancreatic cancer tissues were compared against matched benign pancreatic tissues, and each sample was assessed in triplicate for each miRNA.

**Table 4:** qRT-PCR Primers for miRNAs and U6

miRNA	Primer name	Primer sequence (5' to 3')
miR-15a	RT-primer	GTCGTATCCAGTGCAGGGTCCGAGGTATTCGCACTGGATACGAC CACAAAC
	
	QF	GCGGCTAGCAGCACATAATGG

miR-27a	RT-primer	GTCGTATCCAGTGCAGGGTCCGAGGTATTCGCACTGGATACGAC GCGGAAC
	
	QF	GCGGCTTCACAGTGGCTAAGT

miR-100	RT-primer	GTCGTATCCAGTGCAGGGTCCGAGGTATTCGCACTGGATACGAC CACAAGT
	
	QF	GCGGCAACCCGTAGATCCGAA

miR-125b	RT-primer	GTCGTATCCAGTGCAGGGTCCGAGGTATTCGCACTGGATACGACTCACAAG
	
	QF	GCGGCTCCCTGAGACCCTAAC

miR-181	RT-primer	GTCGTATCCAGTGCAGGGTCCGAGGTATTCGCACTGGATACGAC ACTCACC
	
	QF	GCGGCAACATTCAACGCTGTC

miR-200a	RT-primer	GTCGTATCCAGTGCAGGGTCCGAGGTATTCGCACTGGATACGAC ACATCGT
	
	QF	GCGGCTAACACTGTCTGGTAA

miR-214	RT-primer	GTCGTATCCAGTGCAGGGTCCGAGGTATTCGCACTGGATACGAC ACTGCCT
	
	QF	GCGGCACAGCAGGCACAGACA

miRNA	QR	GTGCAGGGTCCGAGGT

U6	U6QF	CTCGCTTCGGCAGCACA
	
	U6QR	AACGCTTCACGAATTTGCGT
All primers are listed in this table. The RT-primer was used for the reverse transcriptase reaction. QF and QR were used for the PCR reaction. QR was applied to each miRNA test. U6QF and U6QR were used for examination of the U6 gene.		

### Target gene prediction

Target gene prediction was performed to meet the following two criteria. First, miRNA targets were analyzed using following algorithms, TARGETSCAN http://www.targetscan.org/, PICTAR http://pictar.mdc-berlin.de/, TargetBoost, and Miranda (Miranda IM - Home of the Miranda IM client. Smaller, Faster, Easier) and miRBase http://microrna.sanger.ac.uk/sequences/index.shtml. Second, to reduce the likelihood of false positives, only putative target genes predicted by at least two of the programs were accepted.

### Cell proliferation and apoptosis assay

BxPC-3 cells (1 × 10^4 ^per well) were plated in 96-well plates in RPMI medium 1640 and 10% FBS that was supplemented with sodium pyruvate at 37°C in a humidified atmosphere of 5% CO_2_. Cells were transfected with 100 nM miRNA duplex (Ambion) or scrambled duplex (negative control, Ambion) using Lipofectamine 2000 (Invitrogen). For the cell viability study, cytotoxicity was determined in BxCP-3 cells treated with GEM using the CCK-8 assay. Cells were plated at 1 × 10^4 ^per well in a 96-well plate and allowed to adhere for 8 hrs. The cells were then cultured in the absence or presence of 10 μM GEM for 24, 48 or 72 hrs. After GEM treatment, cell viability was measured using the CCK-8 assay.

### Data analysis

Statistical analysis was performed using one-way analysis of variance (ANOVA Dunnett's test) for multiple samples. The paired Student's *t*-test was used to analyze the difference between the control and miRNA-transfected cells. All p-values were obtained using SPSS software, and p-values of <0.05 were considered to be statistically significant.

### Fluorescence reporter construction and luciferase assay

The 3'-untranslated terminal region (3'-UTR) segments (Table 2, Table 3) of 59 bp of the 3'-UTR of the target genes were synthesized by Sangon (Shanghai) and inserted into the psi-CHECK-control vector (Promega) for miRNA functional analysis.

Transient transfection was performed in 293T cells with 100 nM miR-15a or miR-214 mimics and 0.1 μg of psi-CHECK-control or psi-CHECK-3'UTR fluorescence reporter constructs. Fluorescent activities were measured consecutively using Dual-Luciferase assays (Promega) 24 hrs after transfection, according to the instructions of the manufacturer.

## Competing interests

The authors declare that they have no competing interests.

## Authors' contributions

X.J.Z and H.Y contributed equally to this work, performing experiments, analyzing the data, and writing the manuscript; B.H. provided patient samples and clinical data; C.W.Z and H.Z analyzed data and edited the manuscript; Y.Q.C. designed experiments and edited the manuscript. All authors critically reviewed the manuscript.
